# Identification of key residues that regulate the interaction of kinesins with microtubule ends

**DOI:** 10.1002/cm.21568

**Published:** 2019-10-21

**Authors:** Hannah R. Belsham, Claire T. Friel

**Affiliations:** ^1^ School of Life Sciences University of Nottingham, Medical School, QMC Nottingham NG7 2UH United Kingdom

**Keywords:** Kinesin‐1, Kinesin‐13, MCAK, protein engineering, α4 helix

## Abstract

Kinesins are molecular motors that use energy derived from ATP turnover to walk along microtubules, or when at the microtubule end, regulate growth or shrinkage. All kinesins that regulate microtubule dynamics have long residence times at microtubule ends, whereas those that only walk have short end‐residence times. Here, we identify key amino acids involved in end binding by showing that when critical residues from Kinesin‐13, which depolymerises microtubules, are introduced into Kinesin‐1, a walking kinesin with no effect on microtubule dynamics, the end‐residence time is increased up to several‐fold. This indicates that the interface between the kinesin motor domain and the microtubule is malleable and can be tuned to favour either lattice or end binding.

## 1 INTRODUCTION

Kinesins are a large superfamily of microtubule‐associated molecular motors which can be grouped into 17 families based on the sequence of their characteristic motor domain (Lawrence et al., 2004; Wickstead & Gull, 2006). Proteins of the kinesin superfamily are found in all eukaryotes and perform vital roles in the transport of cellular cargo (Hirokawa, Noda, Tanaka, & Niwa, 2009), assembly and action of cilia and flagella (Verhey, Dishinger, & Kee, 2011), development and function of axons (Hirokawa, Niwa, & Tanaka, 2010) and in cell division and chromosome segregation (Cross & McAinsh, 2014).

The kinesin superfamily can be divided into two general classes of activity: translocating kinesins, which move directionally along microtubules, and regulating kinesins, which alter microtubule dynamics, with some families displaying both types of activity. A key feature of the molecular mechanism of kinesins that regulate microtubule dynamics is the ability to recognise the microtubule end. This is demonstrated by an ability to remain at or near the microtubule end for extended times. The Kinesin‐5, Eg5, a microtubule polymerase, resides at microtubule ends for up to 7 s (Chen & Hancock, 2015), and the Kinesin‐8, Kip3, a microtubule depolymerase, resides at the microtubule end for tens of seconds (Su et al., 2011; Varga, Leduc, Bormuth, Diez, & Howard, 2009). Other examples of kinesins with extended microtubule end residence include the Kinesin‐7, CENP‐E (Sardar, Luczak, Lopez, Lister, & Gilbert, 2010), the Kinesin‐4, Kif4 (Subramanian, Ti, Tan, Darst, & Kapoor, 2013), and NOD (Cui et al., 2005), all of which alter microtubule dynamics.

The Kinesin‐13, MCAK, is a microtubule depolymerase and plays a prominent role in regulating microtubule length, in particular during mitosis (Andrews et al., 2004; Domnitz, Wagenbach, Decarreau, & Wordeman, 2012; Li et al., 2016; Maney, Hunter, Wagenbach, & Wordeman, 1998; Shao et al., 2015). In common with other microtubule regulating kinesins, MCAK can distinguish the microtubule end from the lattice (Desai, Verma, Mitchison, & Walczak, 1999) and reside at the microtubule end for up to 15‐fold and on average threefold longer than on the microtubule lattice (Friel & Howard, 2011; Hunter et al., 2003; Patel et al., 2016). By contrast, members of the Kinesin‐1 family are considered purely translocating kinesins and the classic cargo carriers. The translocating activity and cargo carrying function of the Kinesin‐1 family does not require the ability to recognise the microtubule end and this ability has not been detected for a Kinesin‐1. Here we show that the α4‐helix of the kinesin motor domain is a critical region in regulating the balance between microtubule lattice and microtubule end binding. Substitution of Kinesin‐13 family specific residues from the α4‐helix into equivalent positions in a Kinesin‐1 confers the ability to distinguish the microtubule end from the lattice, such that the microtubule end‐residence times of Kinesin‐1 mutants are increased several‐fold.

## 2 RESULTS

### 2.1 Substitution of Kinesin‐13 residues into a Kinesin‐1 motor domain increases microtubule end‐residence time

**Figure 1:**
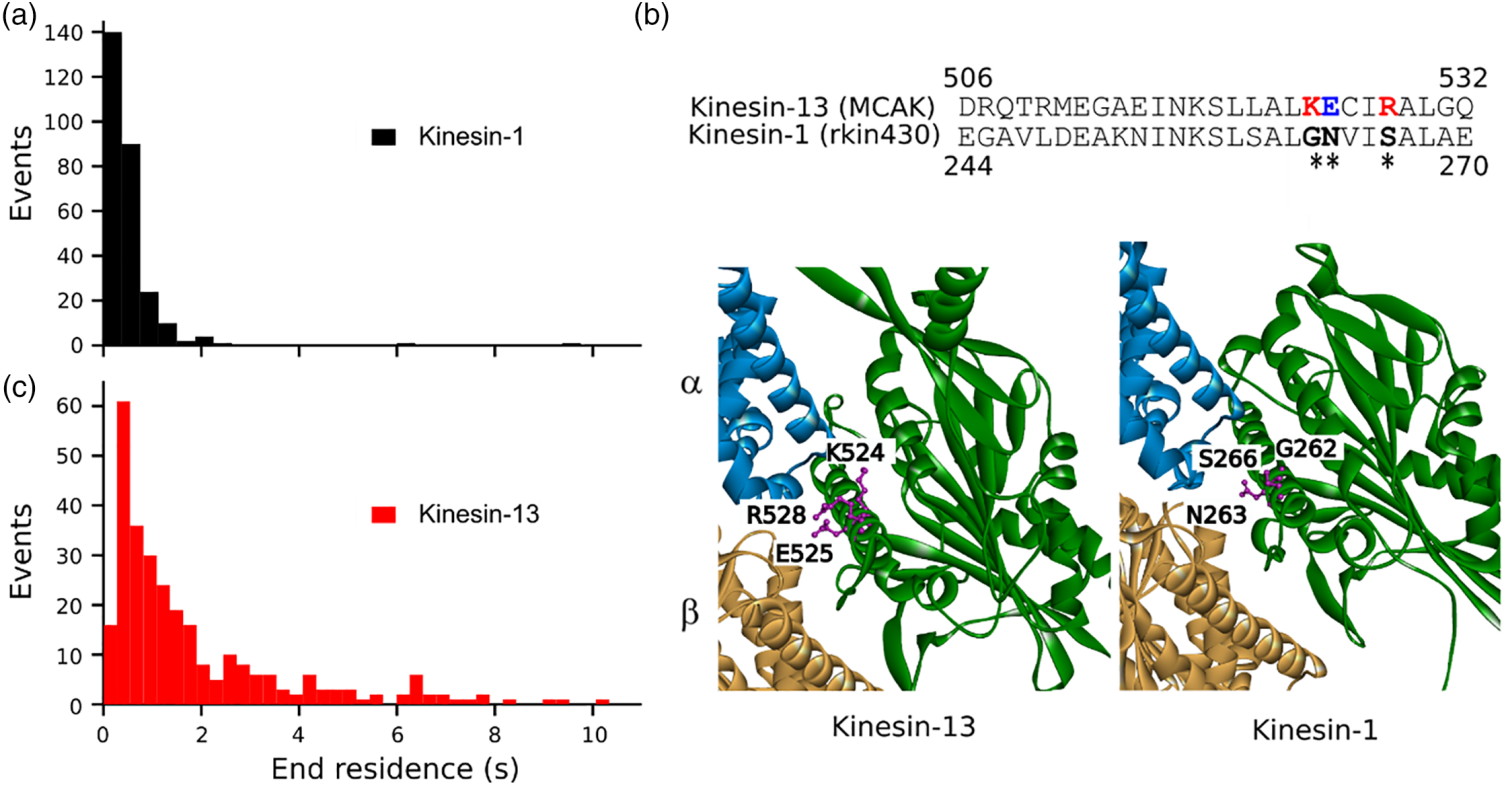
Comparison of microtubule end residence for a purely translocating versus a regulating kinesin. (a) Distribution of microtubule end‐residence times observed for the Kinesin‐1, rkin430 (*n* = 273) and the Kinesin‐13, MCAK (*n* = 289). (b) Sequence alignment of the α4 helix of MCAK and rkin430. Asterisks indicate the positions at which Kinesin‐13 residues were substituted into Kinesin‐1 (red: positive, blue: negative, black: neutral). (c) Structure of MCAK (pdb: 5MIO) and the Kinesin 1, KIF5B (pdb: 4HNA) in complex with tubulin (green: kinesin motor domain, blue: α‐tubulin, orange: β‐tubulin). The substituted residues in the α4 helix are shown in magenta ball and stick format [Color figure can be viewed at http://wileyonlinelibrary.com]

A key characteristic of kinesins that regulate microtubule dynamics is to recognise the microtubule end as distinct from the microtubule lattice. This ability enables regulating kinesins to reside for extended times at microtubule ends and places them at the correct location to influence microtubule growth and/or shrinkage dynamics. The α4‐helix of the motor domain plays a major role in the interface between the kinesin motor domain and the microtubule. Three residues from the α4‐helix of the microtubule depolymerising Kinesin‐13, MCAK, (K524, E525 and R528) are critical to its ability to reside at the microtubule end and essential for depolymerase activity (Patel et al., 2016). To determine if these residues can increase microtubule end binding for a purely translocating kinesin, we substituted the Kinesin‐13 residues into a Kinesin‐1, rkin430. The Kinesin‐1 motor domain has not been shown to recognise the microtubule end and accordingly, we measured an end‐residence time for rkin430 of ≤0.46 ± 0.01 s, which is over four‐fold shorter than MCAK (Figure 1a).

The residues critical to MCAK's microtubule end recognition ability, K524, E525 and R526, correspond by sequence alignment to the rkin430 residues G262, N263 and S266 (Figure 1b) and the structures of the Kinesin‐1 and Kinesin‐13 motor domains confirmed the spatial correspondence of these residues (Figure 1c). We therefore created the rkin430 variants G262K, N263E and S266R to substitute these Kinesin‐13 family specific residues into a Kinesin‐1. We also made a triple mutant containing all three individual mutations.

**Figure 2:**
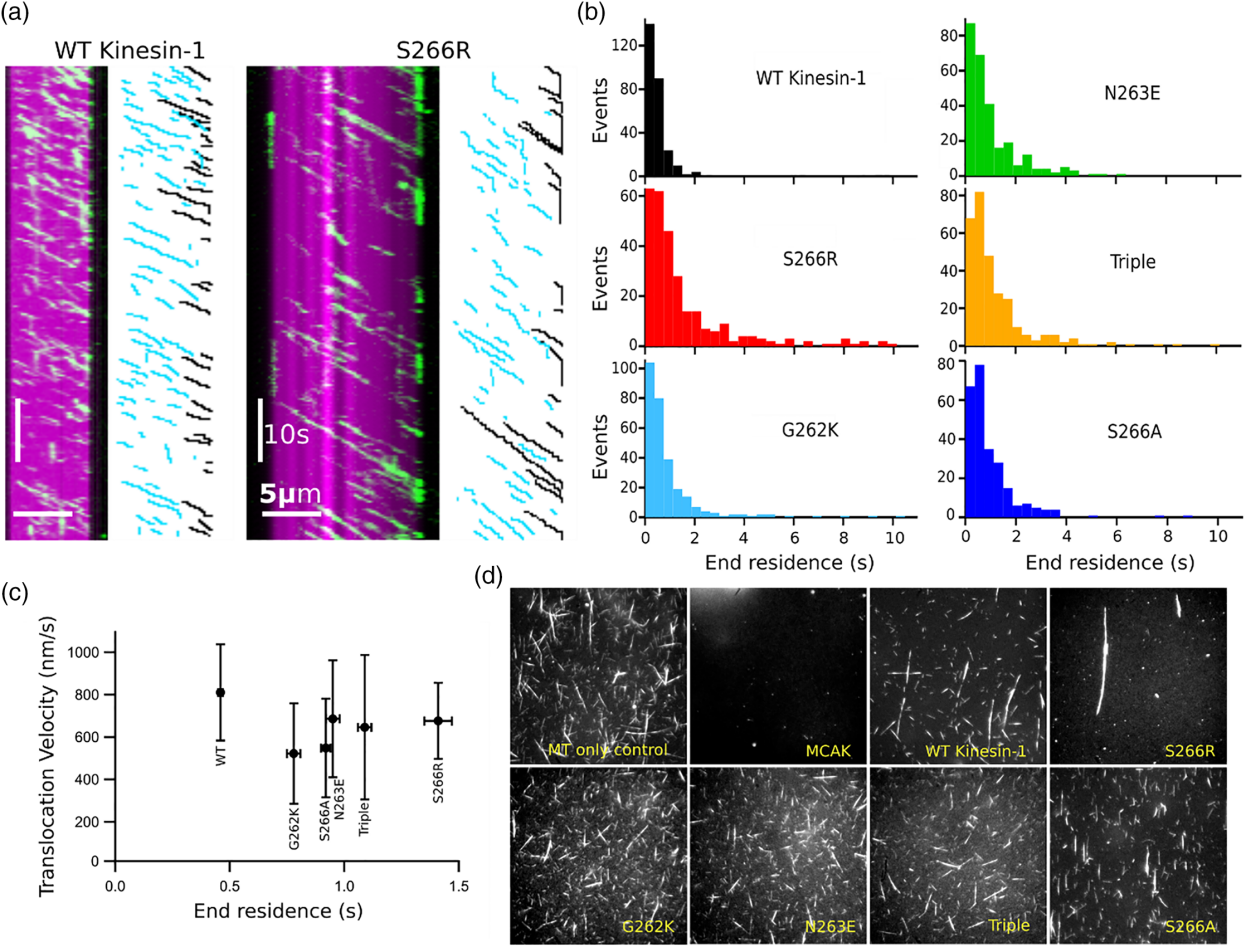
Substitution of Kinesin‐13 residues into the α4 helix of Kinesin‐1 increases its microtubule end residence. (a) Kymographs showing the interaction of the Kinesin‐1, rkin430 (green) and the Kinesin‐1 variant S266R (green) with a microtubule (magenta). Schematic highlighting kinesin interaction events is shown alongside each kymograph: events contained within the microtubule (blue) and events that reach the microtubule end (black). (b) Distribution of microtubule end‐residence times for wild‐type Kinesin‐1 and the variants S266R, G262K, N263E, a triple mutant (G262K/N263E/S266R) and S266A. (c) Relationship between translocation velocity and end residence for rkin430 and all variants. (d) End point of incubation of MCAK, rkin430 and variants of rkin430 with fluorescently labelled microtubules [Color figure can be viewed at http://wileyonlinelibrary.com]

All four Kinesin‐1 variants had a significantly longer microtubule end‐residence time than wild‐type Kinesin‐1: 0.78 s, 0.95 s, 1.41 s, and 1.09 s for G262K, N263E, S266R and the triple mutant, respectively (*p* < 0.001, Kolmogorov–Smirnov test) (Table 1 and Supporting Information Figure S1a). Increased residence times resulting from these substitutions are specific to the microtubule end: lattice residence was either decreased or unaffected for each of these kinesin‐1 variants (Supporting Information Table S1). The substitution S266R had the largest effect on end‐residence time, causing a ≥ three‐fold increase relative to wild‐type Kinesin‐1. Kymographs show that S266R stays on the microtubule end for an average of 5 frames (5 pixels in the vertical) compared to only a single frame for wild‐type Kinesin‐1 (Figure 2a). The distribution of microtubule end‐residence times for each variant shows that increased microtubule end residence resulted from an increased proportion of longer end residence events rather than a general increase in the duration of all end binding events (Figure 2b).

**Table 1:** Compiled data for microtubule end residence, velocity, run length, ATPase rate and depolymerisation activity for WT‐rkin430 and variants

	WT	G262K	N263E	S266R	Triple	S266A	MCAK^a^
End residence (s)	0.46 ± 0.01 (*n* = 273)	0.78 ± 0.03 (*n* = 285)	0.95 ± 0.03 (*n* = 272)	1.41 ± 0.06 (*n* = 284)	1.09 ± 0.03 (*n* = 296)	0.92 ± 0.02 (*n* = 252)	2.03 ± 0.13 (*n* = 238)
Basal ATPase rate (s^−1^)	0.14 ± 0.07 (*n* = 3)	0.11 ± 0.06 (*n* = 3)	0.18 ± 0.13 (*n* = 3)	0.11 ± 0.08 (*n* = 3)	0.23 ± 0.20 (*n* = 3)	0.24 ± 0.06 (*n* = 3)	0.0021 ± 0.0003 (*n* = 4)
Velocity (nm/s)	810 ± 227 (*n* = 382)	522 ± 237 (*n* = 363)	686 ± 276 (*n* = 342)	676 ± 179 (*n* = 375)	646 ± 341 (*n* = 363)	548 ± 223 (*n* = 367)	n/a
Run length (μm)	3.06 ± 1.16 (*n* = 382)	1.29 ± 0.49 (*n* = 363)	1.05 ± 0.39 (*n* = 342)	1.54 ± 0.89 (*n* = 375)	0.92 ± 0.38 (*n* = 363)	1.48 ± 0.99 (*n* = 367)	n/a
Depolymerisation activity	−	−	−	−	−	−	+++

a Data from Patel et al. (2016).

### 2.2 Increased end residence is not due to misfolding or disruption of translocation activity and is specifically favoured by Kinesin‐13 residues

To determine whether the amino acid substitutions had a deleterious effect on the functionality of the Kinesin‐1 motor domain, we measured the ability of each mutant to turnover ATP. The basal ATPase rate was not significantly different to wild type for any of the mutants (Table 1) and there was no correlation between ATPase rate and microtubule end‐residence time (Supporting Information Figure S1b). The ability of the Kinesin‐1 mutants to turnover ATP at an equivalent rate to wild type indicates that the motor domain is correctly folded and functional.

Each of the amino acid substitutions caused a moderate reduction in translocation velocity (Table 1). However, there was no relationship between translocation velocity and end‐residence time (Figure 2c). This indicates that the increased end‐residence times do not simply result from slower translocation velocities causing the mutants to take longer to move over the microtubule end. Taken together, these data indicate that substitution of Kinesin‐13 specific residues into the α4 helix of Kinesin‐1 caused a specific increase in microtubule end‐residence time.

To establish whether the observed increased end‐residence times were specifically due to introduction of Kinesin‐13 residues rather than loss of Kinesin‐1 residues, we created the rkin430 variant S266A. The microtubule end‐residence time for this variant was increased relative to wild‐type Kinesin‐1 (Table 1 and Figure 2b) but not to the same degree as the increase caused by introduction of the Kinesin‐13 family specific residue at this position. The end residence of S266A was 0.9 s compared to 1.4 s for the Kinesin‐13 specific substitution, S266R. These data suggest not only that the Kinesin‐13 residue at this position specifically favours microtubule end binding over lattice binding, but also that the Kinesin‐1 residue disfavours binding at the microtubule end.

### 2.3 Increased microtubule end residence alone does not confer depolymerase activity

The ability to recognise the microtubule end is a property that distinguishes purely translocating kinesins from regulating kinesins and microtubule end recognition is a critical part of the molecular mechanism of the microtubule depolymerising Kinesin‐13, MCAK. To establish whether increased microtubule end residence is sufficient to confer depolymerase activity, we determined the effect of the Kinesin‐1 variants on microtubule stability by two different methods. When fluorescently labelled microtubules were incubated with wild‐type Kinesin‐1 or any of the Kinesin‐1 variants no significant depolymerisation of microtubules was observed (Figure 2d). The appearance of fluorescently labelled microtubules after incubation with Kinesin‐1 or variants was not significantly altered, except in the case of S266R which appears to have a microtubule bundling effect. By contrast, under the same conditions microtubules were completely depolymerised by MCAK. The lack of depolymerisation activity of these kinesin‐1 variants was also demonstrated using a light scattering assay in which the presence of microtubule is detect by their ability to scatter light at 350 nm (Supporting Information Figure S2). No significant change in light scattering signal was observed for Kinesin‐1 and variants, whilst the Kinesin‐13, MCAK, caused a large decrease in light scatter due to depolymerisation of microtubules. There is a small but significant decrease in the change in light scatter for WT Kinesin‐1 relative to a microtubule only sample (*p* =0 .02). This may be due to stabilisation of microtubules by Kinesin‐1 binding. No significant difference is observed for Kinesin‐1 variants indicating neither a stabilising nor destabilising effect on microtubules.

## 3 DISCUSSION

Despite high sequence and structural conservation of the superfamily defining motor domain, kinesins from different families display a remarkable diversity of kinetic and functional properties (Cross & McAinsh, 2014; Hirokawa et al., 2009; Peterman & Scholey, 2009). A key property of kinesins that regulate microtubule dynamics, such as the Kinesin‐13 family of microtubule depolymerases, is their ability to recognise the microtubule end. To better understand microtubule end recognition by kinesins, we introduced Kinesin‐13 family specific residues into the motor domain of a Kinesin‐1 rkin430. Each of the Kinesin‐1 variants G262K, N263E and S266R, increased the microtubule end‐residence time with the largest effect caused by substitution of serine by arginine at position 266. Interestingly, the effect of introducing all three mutations in a triple mutant construct was not additive. Rather, the behaviour of a triple mutant was similar to the single mutant N263E suggesting that this position on the tubulin binding face of the motor domain is pivotal to the balance between microtubule lattice and microtubule end affinity. In a structure of Kinesin‐1 in complex with the microtubule lattice conformation of the α/β‐tubulin heterodimer (Shang et al., 2014), the N263 side chain points towards the binding interface, which may explain its dominance. By contrast, the S266 side chain is oriented parallel to the tubulin binding face, which could explain why mutation of this residue has a pronounced effect on end binding whilst having little effect on translocation activity. The residue at this position may become more important in an interaction with a curved tubulin conformation likely found at the microtubule end.

**Figure 3:**
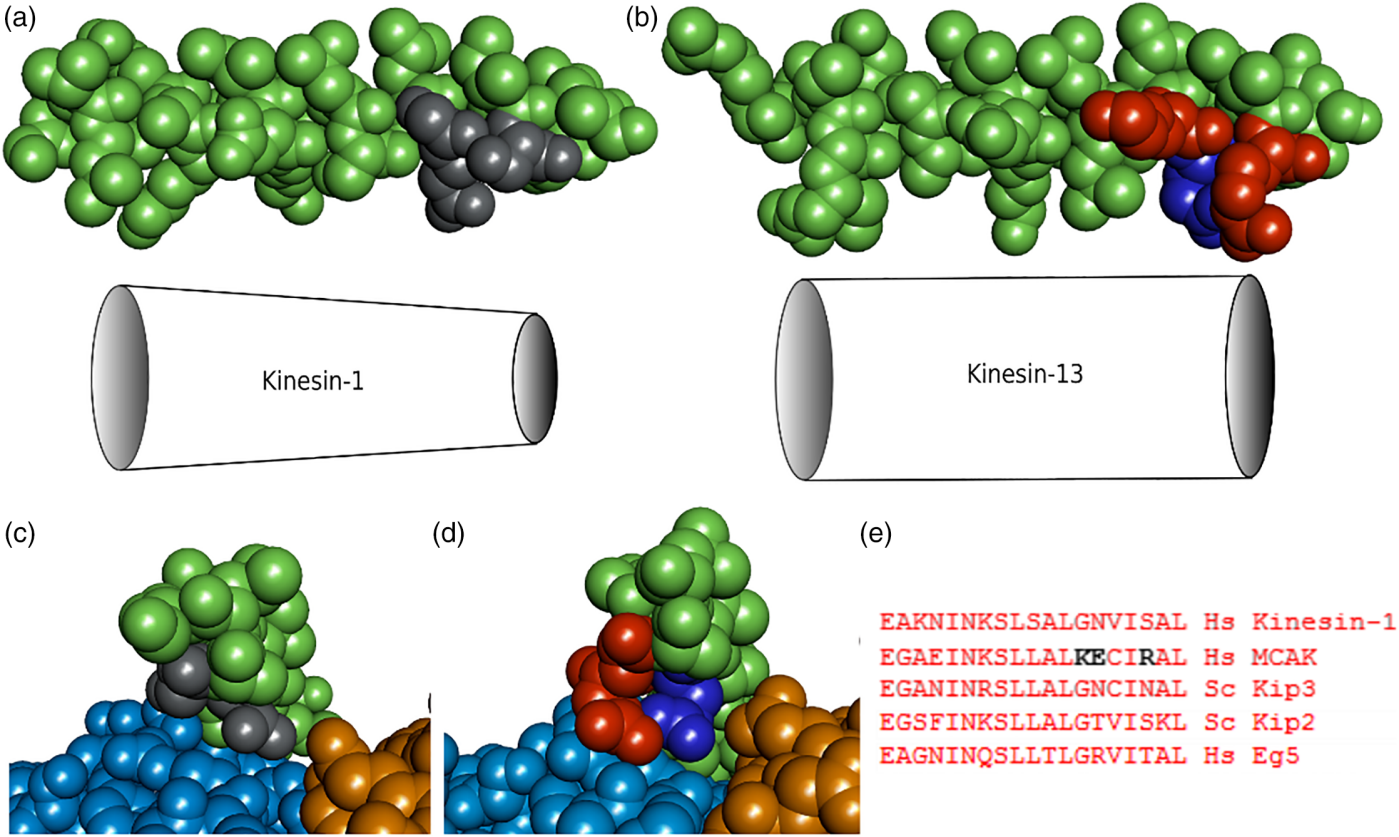
C‐terminus of Kinesin‐13 α4‐helix is bulkier than Kinesin‐1. (a and b) Space‐fill models of the α4‐helix of (a) Kinesin‐1 (pdb: 4HNA) and (b) Kinesin‐13 (pdb: 5MIO). The Kinesin‐1 α4‐helix tapers towards the C‐terminus, whereas the Kinesin‐13 α4‐helix remains bulky. (c and d) End on view of the C‐terminus of the α4‐helix of (c) Kinesin‐1 and (d) Kinesin‐13 in complex with α/β‐tubulin. Kinesin (green), α‐tubulin (light blue), β‐tubulin (orange). The critical kinesin residues mutated in this study are coloured grey (neutral), red (positive charge) or dark blue (negative charge). (e) Alignment of the C‐terminal end of the α4‐helix for Kinesin‐1, MCAK (Kinesin‐13) and three other kinesins that regulate microtubule dynamics [Color figure can be viewed at http://wileyonlinelibrary.com]

The Kinesin‐13 family specific residues K524, E525 and R528 are not only charged but also larger than the equivalent Kinesin‐1 residues. The impact of this is to create a bulkier α4‐helix (Figure 3a,b) in the Kinesin‐13 motor domain relative to Kinesin‐1. The Kinesin‐1 α4‐helix forms a cone‐like shape, with the diameter at the C‐terminal end being smaller than at the N‐terminal end (Figure 3a), whereas the Kinesin‐13 α4‐helix is more cylindrical due to the presence of these bulkier family specific residues at the C‐terminus (Figure 3b). Structures of the kinesin motor domain in complex with tubulin (Gigant et al., 2013; Wang et al., 2017) show that the α4‐helix contacts the α/β‐tubulin heterodimer at the interface between the α and β subunits, the so‐called “intradimer groove” (Figure 3c,d). The bulky nature of the Kinesin‐13 residues may enhance sensing of tubulin curvature by responding to the reduction of space at the interface between the α and β subunits in a curved tubulin conformation such as that proposed to form at the microtubule end compared to a straight tubulin conformation found within the microtubule lattice.

Whilst introduction of the Kinesin‐13 residues into the Kinesin‐1 motor domain increased microtubule end‐residence time relative to wild type, none of the variants had significant depolymerisation activity. The end‐residence times for the Kinesin‐1 variants are shorter than for the specialist microtubule depolymerising kinesin, MCAK, and it is possible that a longer microtubule end residence is required for depolymerase activity. However, it is more likely that structural elements in addition to the α4 helix, such as the Kinesin‐13 specific extended Loop 2, are also required for depolymerisation activity (Ogawa, Nitta, Okada, & Hirokawa, 2004; Patel et al., 2016; Shipley et al., 2004). It is possible that the α4‐helix is principally a curvature sensing region, which is required to bind at the microtubule end, whilst the region of the Kinesin‐13 motor domain that actively removes tubulin causing depolymerisation is located elsewhere.

Alignment of the α4‐helix from other kinesins that regulate microtubule dynamics, such as the microtubule depolymerising yeast kinesin, Kip3 and the microtubule polymerising kinesins Kip2 and Eg5, do not show any similarity of sequence to the Kinesin‐13 family specific residues at the positions studied here (Figure 3e). It is likely that other microtubule regulating kinesin use different regions of the motor domain to recognise the microtubule end. In the case of the microtubule depolymerising kinesin Kip3, Loop 11 which connects with the N‐terminal end of the α4‐helix has been shown to be the critical region for microtubule end binding (Arellano‐Santoyo et al., 2017). Substituting Loop 11 of Kip3 into a Kinesin‐1 increases the microtubule end‐residence time comparable to that of Kip3. However, similar to our observations here, other regions of the motor domain are also required to confer significant depolymerisation activity.

Here we show that the nature of the C‐terminal end of the α4‐helix is critical to the microtubule end recognition ability of the Kinesin‐13 family of microtubule depolymerases. Transplanting the Kinesin‐13 specific residues found here into an alternative kinesin motor confers the ability to recognise the microtubule end. This study highlights the potential to tune the motor domain‐tubulin interface to favour either microtubule lattice or end binding, which has implications for our understanding of the molecular mechanism of kinesins with both translocating and regulating activity and for our ability to manipulate the function of the kinesin motor domain.

## 4 MATERIALS AND METHODS

### 4.1 Preparation of kinesin

Rat kinesin‐1, rkin430‐GFP‐h6, and all variants were expressed in BL21 *E. coli* cells. The bacterial pellet was lysed in 50 mM sodium phosphate pH 7.5, 100 mM NaCl, 1 mM MgCl_2,_ 10 μM ATP, 5 mM β‐mercaptoethanol, with EDTA‐free protease inhibitor cocktail tablet (Roche) using a cell disrupter at 35kpsi. Kinesin‐1 and variants were purified by anion exchange, followed by Ni‐affinity chromatography (Rogers et al., 2001). Full‐length, human MCAK‐his6 was expressed in Sf9 cells and purified using cation exchange, followed by Ni‐affinity chromatography (Helenius, Brouhard, Kalaidzidis, Diez, & Howard, 2006).

### 4.2 Microtubules

Porcine brain tubulin was purified from homogenised brain tissue using the high ionic strength method (Castoldi & Popov, 2003). Fluorescently labelled microtubules were prepared as described previously (Patel et al., 2016).

### 4.3 Single molecule TIRF assays

Single molecules of rkin430‐GFP and variants were observed on immobilised, GMPCPP‐stabilised, 25% rhodamine‐labelled microtubules in BRB12 (12 mM PIPES/KOH pH 6.9, 1 mM EGTA, 2 mM MgCl_2,_) plus 1 mM ATP, 0.1% Tween 20, 0.1 mg/ml BSA and antifade (1% 2‐mercaptoethanol, 40 mM glucose, 40 mg/ml glucose oxidase, 16 mg/ml catalase) using TIRF microscopy (Patel et al., 2016). Images were collected at a frame rate of 2.7 Hz. Time spent by single kinesin molecules at the microtubule end and on the microtubule lattice was measured in FIJI (Schindelin et al., 2012) using kymographs of individual microtubules. The microtubule end was defined as the final pixel at either end of a microtubule in the rhodamine channel with a fluorescence signal above the background subtracted threshold. An event was defined as a pixel containing fluorescence intensity above background in the GFP channel. Events were considered discrete when separated by ≥1 non‐event pixel in either the vertical (time) or horizontal (distance) axis. Translocating events were defined as events which moved in a unidirectional manner for at least 3 pixels in the vertical (>750 ms). When events crossed and it was not possible to identify individual events, they were discounted from the analysis. All other events were captured and classified.

### 4.4 ATPase assays

Reactions were initiated by addition of 1 μM Kinesin to BRB12 buffer containing 2 mM ATP and samples taken every 5 min, quenched with an equal volume of ice‐cold 0.6 M perchloric acid, neutralised with Tris/KOH and clarified by centrifugation. The progression of the reaction was monitored by separating ADP from ATP by HPLC (Friel, Bagshaw, & Howard, 2011).

### 4.5 Depolymerisation assays

GMPCPP‐stabilised, rhodamine‐labelled microtubules were incubated with 40 nM kinesin and 1 mM ATP in BRB12 for 20 min. The reactions were then flowed into channels made from poly‐lysine coated cover glasses and imaged. Microtubule depolymerisation was also monitored by light scattering at 350 nm. Kinesin protein was added to microtubules at final concentrations of 50 nM and 1 μM, respectively, in the buffer BRB80 pH 6.9, 75 mM KCl, 1 mM MgATP, 1 mM DTT, 200 μg/ml BSA. The light scatter signal was recorded at 5 s intervals using a Hitachi F2500 fluorimeter.

## AUTHOR CONTRIBUTIONS

H.R.B. and C.T.F. designed the experiments. H.R.B. performed the experiments and analysed the data. H.R.B. and C.T.F. interpreted the results and wrote the article.

## Supporting information


**Data S1** Table S1, Figures S1 and S2.

## Data Availability

The data that support the findings of this study are available from the corresponding author upon reasonable request.
