# Supplementary material for: Identification of key residues that regulate the interaction of kinesins with microtubule ends
Source: Cytoskeleton (Hoboken). 2019 Oct 21;76(7-8):440–6. doi: 10.1002/cm.21568 (PMC6899999; doi:10.1002/cm.21568)
Supplement: Supplementary file 1 — Data S1 Table S1, Figures S1 and S2. [file CM-76-440-s001.docx]

### Supplementary Information

**Table S1:** Microtubule interaction parameters determined for rkin430 and variants from data acquired using single molecule TIRF. Errors are standard deviation. *k_off_* end and *k_off_* lattice were determined from the fit of the cumulative distribution of microtubule end and microtubule lattice residence times, respectively, to a single exponential function (SI Fig S1b). *k_on_* was calculated as described in Helenius *et al* (2006) Nature **441**: 115-119. The velocity and run length were determined only for events classed as translocating. **^†^**n is number of microtubules, in all other cases n is the number of events.

|  | WT | G262K | N263E | S266R | Triple | S266A |
| --- | --- | --- | --- | --- | --- | --- |
| *k_off_* end (s^-1^) | 2.18±0.03  (n=273) | 1.28±0.02  (n=285) | 1.05±0.01  (n=272) | 0.71±0.01  (n=284) | 0.92±0.01  (n=296) | 1.09±0.01  (n=252) |
| *k_off_* lattice (s^-1^) | 0.44±0.01  (n=194) | 0.54±0.01  (n=207) | 0.80±0.02  (n=232) | 0.44±0.01  (n=203) | 0.61±0.02  (n=239) | 0.38±0.02  (n=249) |
| *k_on_* ^†^  (µm^-1^ nM^-1^ s^-1^) | 0.05±0.40  (n=5) | 0.11±0.17  (n=4) | 0.18±0.16  (n=4) | 0.08±0.22  (n=5) | 0.14±0.24  (n=5) | 0.06±0.28  (n=7) |
| % events translocating | 48 | 38 | 25 | 55 | 23 | 30 |
| % events diffusive | 20 | 34 | 38 | 14 | 51 | 33 |

**
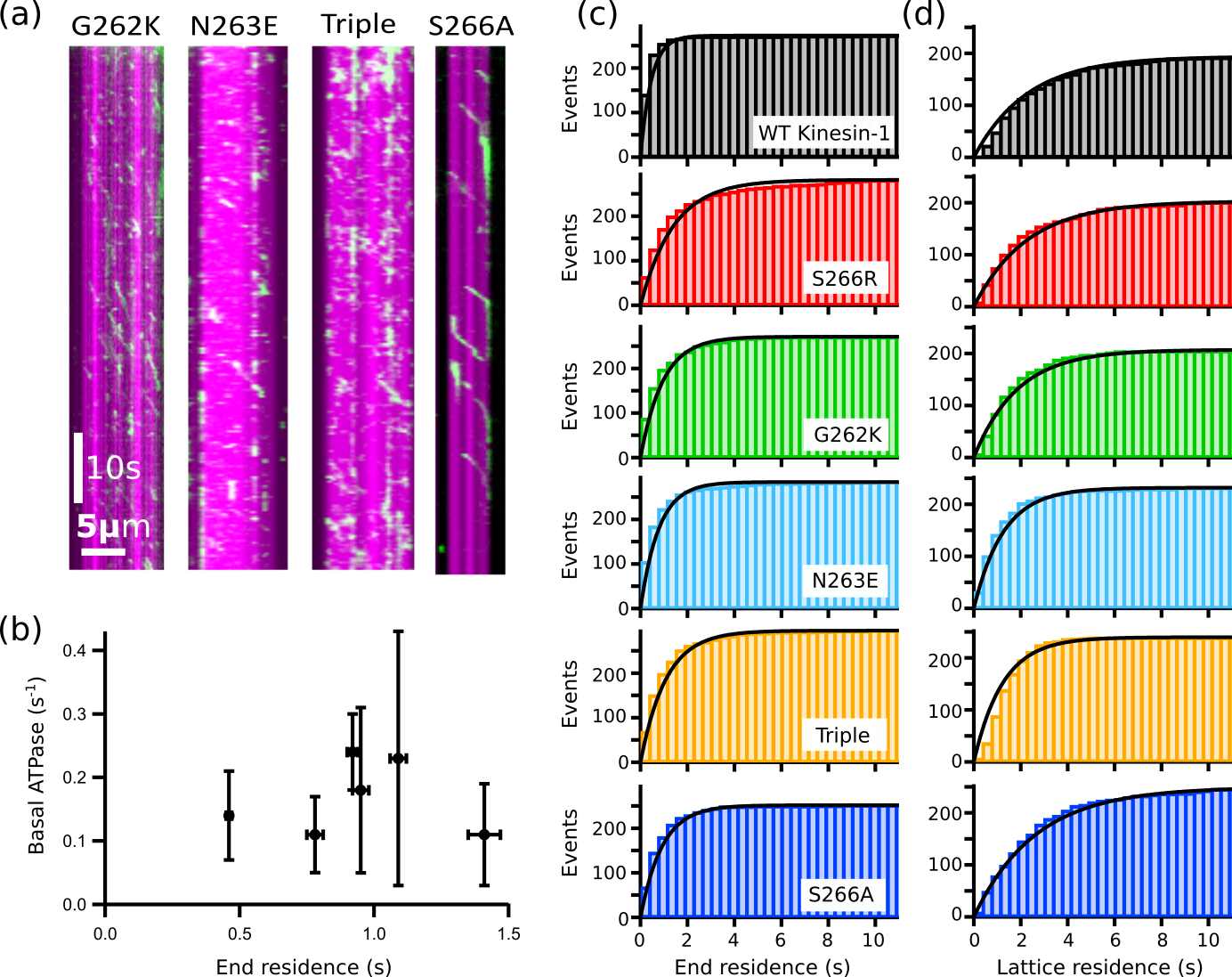
**

**Figure S1:** (a) Kymographs showing the interaction of GFP-tagged rkin430 variants (green), with GMPCPP-stabilised, rhodamine labelled microtubules (magenta). (b) Relationship between microtubule end residence and basal ATPase rate. (c) Cumulative histograms of microtubule end residence events. (d) Cumulative histograms of microtubule lattice residence events. (c & d) Each data set is fit to a single exponential function (black).

**
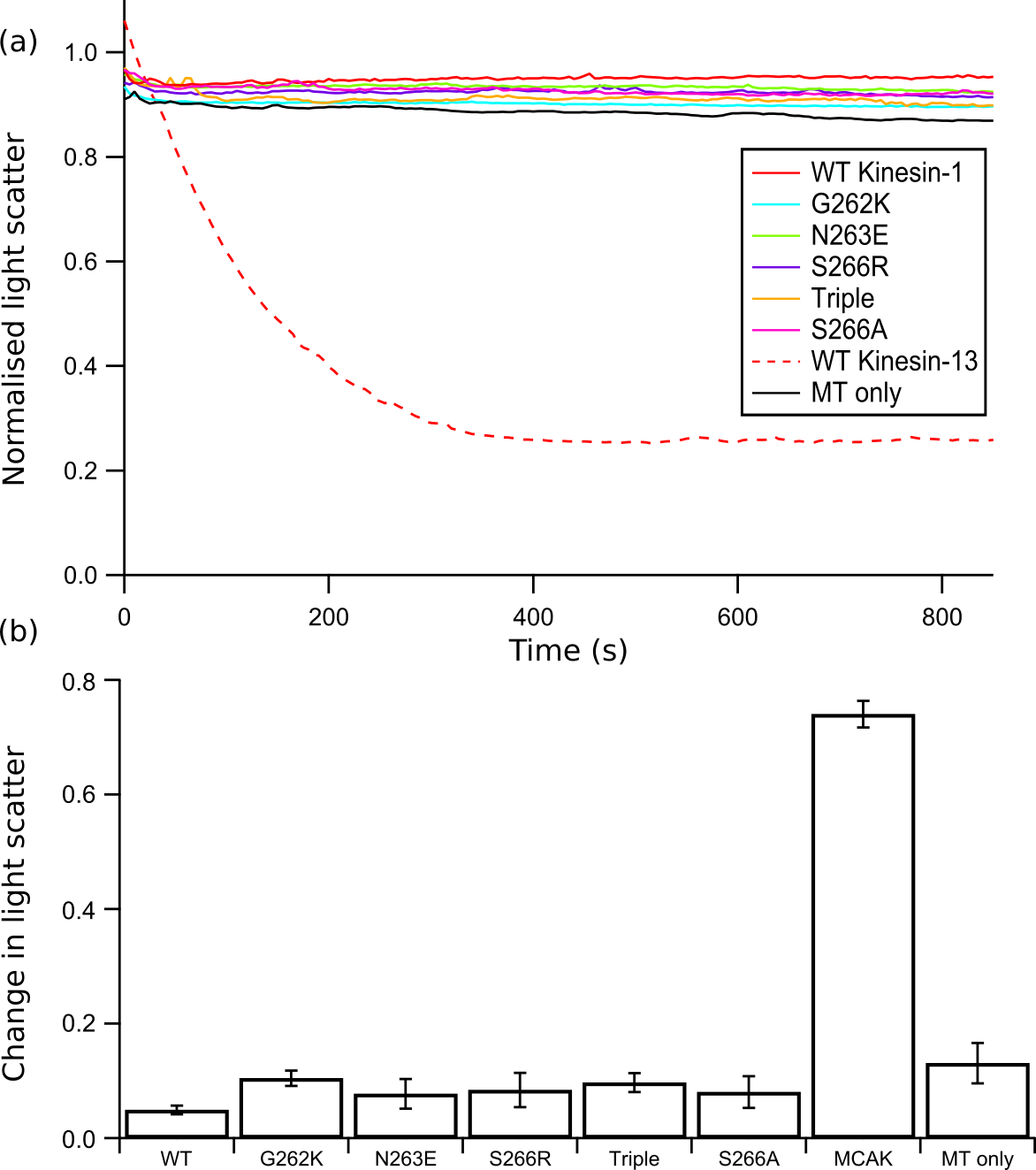
**

**Figure S2:** (a) Normalised light scatter at 350 nm of microtubules over time. Kinesin is added at time zero. (b) Change in light scatter between signal prior to addition of kinesin and t = 800 s. A large change in light scatter indicates loss of microtubules due to depolymerisation activity. The positive control WT Kinesin-13 (MCAK) shows high microtubule depolymerisation activity. None of the Kinesin-1 variants show any significant depolymerisation activity (p = 0.12 – 0.29) with the change in signal being similar to that of a microtubule sample (buffer added at t = 0).
